# Exploring the Impact of Traditional Practices on *Vibrio cholerae* Outbreaks in Rural Nigerian Communities: A Field Study with Educational and Behavioral Interventions

**DOI:** 10.3390/ijerph22040483

**Published:** 2025-03-24

**Authors:** Ijebusonma Agundu, Olalekan Oluwayomi, Tim Ford

**Affiliations:** 1 Department of Public Health, University of Massachusetts Lowell, Lowell, MA 01854-5127, USA; 2 Allen Foundation, Lagos 100001, Nigeria; 3 Department of Biomedical and Nutritional Sciences, University of Massachusetts Lowell, Lowell, MA 01854-5125, USA

**Keywords:** cholera, waterborne disease, sanitation, community engagement, WaSH, underserved communities

## Abstract

This study examined the link between traditional practices, water stewardship, and cholera outbreaks in three rural Nigerian communities (Enugu, Delta, and Ondo States) in 2020. A sample of 180 participants, representing different socio-economic backgrounds, was surveyed using a mixed-methods approach. Knowledge-based pre-test and post-test measures were employed to assess changes in the understanding of cholera transmission, prevention, and water infrastructure. Chi-square and logistic regression analyses were applied to examine the relationship between socioeconomic status, trust in traditional water sources, and cholera knowledge. Educational seminars were conducted, followed by six months, before administering the post-test to the same population. Key findings revealed that 47% of respondents washed animals in water sources, 42% did not treat their water, and 53% were unaware of cholera-reporting practices. The post-test results showed that 80% of participants could correctly identify cholera symptoms following educational interventions (*p* < 0.001). Water, sanitation, and hygiene (WaSH) program awareness was significantly associated with reduced cholera incidence (*p* = 0.005), while certain cultural practices, such as washing slaughtered animals in main water sources, were associated with increased cholera (*p* < 0.002). This study highlights the need for increased awareness of source water quality, better stewardship, and trust-building efforts to provide culturally appropriate interventions in mitigating these outbreaks.

## 1. Introduction

Cholera, an acute diarrheal disease caused by *Vibrio cholerae*, continues to present a significant public health challenge, particularly in regions with fragile healthcare systems and inadequate access to clean water and sanitation [1]. Despite being preventable through improved water, sanitation, and hygiene (WaSH) interventions [2,3,4], cholera is associated with morbidity and mortality in many developing countries. This is especially true in sub-Saharan Africa, where Nigeria, one of the most affected countries in the region, has experienced recurrent cholera outbreaks with over 93,000 cases and 3298 deaths reported in 2020 alone [5]. The surge in cases during this period coincided with the global COVID-19 pandemic, which placed additional strain on Nigeria’s already overburdened healthcare infrastructure, and removed resources critical for cholera surveillance and response [6]. Cholera claimed 3604 lives in Nigeria in 2021 [5], a death toll that surpassed that of COVID-19 in the same year. This underscores the critical, yet often underappreciated, public health threat posed by waterborne diseases in the region.

The burden of cholera is disproportionately borne by the poorest and most marginalized populations, particularly in areas where water treatment is inadequate, sanitation infrastructure is poor, and hygiene education is limited [7,8]. Cholera outbreaks are particularly prevalent in countries like Nigeria, where traditional water sources such as rivers, wells, and open springs are often contaminated due to practices like open defecation, washing of animals near water sources, and the lack of effective waste disposal systems [9]. These practices, rooted in cultural habits and limited resources, significantly increase the risk of cholera transmission. Similar patterns have been observed across East Africa, where studies from Tanzania and other countries indicate that inadequate water management and open defecation are key drivers of cholera outbreaks [10,11].

In addition to environmental factors, cultural beliefs and societal norms play a crucial role in shaping health behaviors and attitudes toward cholera prevention. In many Nigerian communities, there is widespread mistrust of government health interventions and modern water treatment technologies, such as chlorination. This mistrust is often compounded by a limited understanding of the disease’s causes and transmission mechanisms. Some communities attribute cholera to supernatural forces, which complicates efforts to promote prevention measures [12]. These socio-cultural factors, inadequate government responses, and poor sanitation infrastructure create significant barriers to effective cholera control.

Addressing cholera in Nigeria requires a multifaceted approach that goes beyond the technical aspects of water treatment and infrastructure improvement. Enhancing community engagement, promoting hygiene education, and addressing cultural beliefs are essential to any effective cholera control strategy. This study aims to explore the role of cultural practices and beliefs in the persistence of cholera outbreaks in Nigeria. Specifically, it examines three states—Enugu, Delta, and Ondo—to understand local knowledge of cholera transmission, the use of water sources, and hygiene behaviors. By using a socio-ecological framework [13], this study seeks to identify culturally sensitive, community-driven strategies for improving cholera prevention. Such an approach recognizes the importance of integrating local knowledge, cultural beliefs, and practices into public health interventions, ensuring they are more relevant, acceptable, and sustainable [14,15].

This work underscores the need for a comprehensive approach that not only tackles the environmental determinants of cholera but also addresses the social, cultural, and behavioral factors that perpetuate its transmission. By focusing on these dimensions, the study seeks to contribute to developing more effective, locally tailored public health interventions, ultimately aiming to reduce cholera incidence in Nigeria and other cholera-endemic regions worldwide.

## 2. Materials and Methods

### 2.1. Study Design

**Figure 1:**
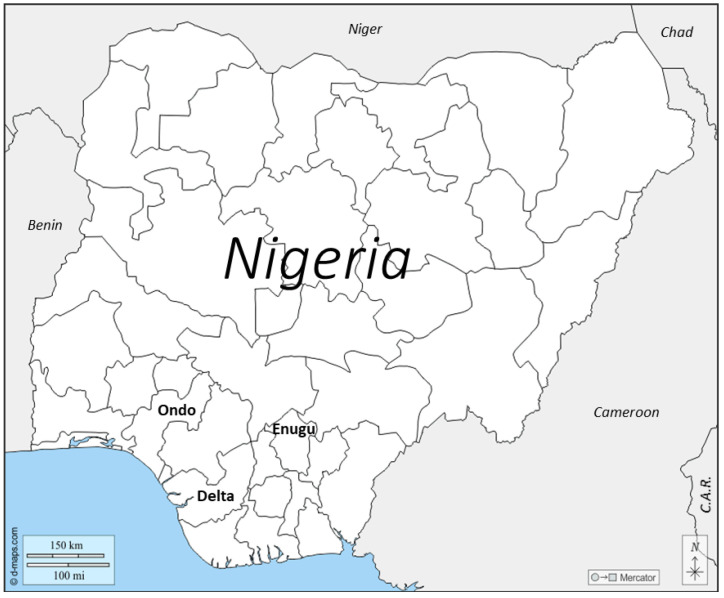
Map of Nigeria on the West Coast of Africa showing position of Ondo, Delta, and Enugu States. Adapted from https://d-maps.com/carte.php?num_car=4864&lang=en (accessed on 9 February 2025).

This study employed a quasi-experimental mixed-methods design, including both pre- and post-test assessments, to evaluate the effectiveness of educational interventions aimed at reducing cholera outbreaks in three rural Nigerian communities (Ugbwaka in Enugu, Ikare in Ondo, and Ogwashi-Ukwu in Delta States) (Figure 1). The mixed-methods approach used here is particularly useful in understanding complex social behaviors and health outcomes and has been widely employed in cholera education and research [16,17].

Communities in each of the three states—Ogwashu (Delta), Ikare (Ondo), and Ugbwaka (Enugu)—were selected based on their cholera incidence rates, representing areas with varying exposure to cholera risk and objectively defined for this study as high (recurrent outbreaks), mixed, and low risk (Table 1).

#### 2.1.1. Sampling Strategy

The sampling strategy employed in this study involved stratified purposive sampling, a technique designed to target specific subgroups within the population based on predetermined characteristics relevant to the research objectives. This approach ensured representation of communities with varying cholera outbreak frequencies, including high-risk areas like Ogwashu (Delta) with recurrent outbreaks.

##### Stratification Criteria and Vulnerability Characteristics

The study focused on communities with varying levels of risk for cholera (Table 1). These risk categories were primarily based on prior cholera incidence data, provided by the Nigerian Centre for Disease Control and Prevention (NCDC) [18], geographical location, and consultation with local health authorities and the Allen Foundation (Supplemental Material, Box S1). Incidence was based on symptomatic data obtained by the NCDC and likely underestimates actual numbers, as has been shown in other studies [19]. It should also be noted that mild cholera can be indistinguishable from other diarrheal diseases, which would tend to overestimate these numbers [8]. Thirty volunteers from the Allen Foundation played a crucial role in data collection, facilitating surveys and focus group discussions within the local communities. These volunteers were trained to administer the survey questionnaires and assist with organizing the focus group discussions. Their local knowledge and familiarity with the community helped to ensure that data collection was both efficient and culturally sensitive, ultimately contributing to the accuracy and reliability of the study’s findings. This community engagement strategy was essential in gathering comprehensive data from a diverse range of participants, ensuring the robustness of the study’s results.

The vulnerability criteria for selection within these risk categories included the following:

- Water Quality and Sanitation Infrastructure: Communities with inadequate access to safe drinking water or sanitation facilities were deemed more vulnerable to cholera transmission.
- Socioeconomic Status: Lower-income communities with limited access to health services or hygiene education were prioritized for intervention.
- Cultural Practices: Communities with prevalent traditional practices, such as washing animals in communal water sources or open defecation, were included due to the heightened risk these practices pose for cholera transmission.

##### Purposive Selection Within Strata

Purposive selection aimed to ensure that participants were representative of the key characteristics of vulnerability within each risk category. Specifically, communities with a high likelihood of cholera transmission and a demonstrated need for hygiene interventions were selected. The focus was on recruiting participants from areas where cholera prevention efforts could have the most significant impact rather than achieving a perfectly representative sample.

##### Opportunistic Sampling of Sites

While the study targeted specific communities based on cholera risk, it should be noted that some sites were opportunistically sampled based on availability and collaboration with local stakeholders, including the Allen Foundation. The Foundation’s involvement in selecting communities ensured that the study focused on those with the highest need for intervention while also aligning with logistical and ethical considerations.

The full intervention could not be implemented in Delta due to logistical challenges, including limited access to remote areas and insufficient infrastructure for widespread WaSH programs. This was reflected in reduced improvements in cholera prevention relative to the other states (see Results and Discussion). In Ondo, the intervention was only partially implemented, due to resource constraints and timing issues, preventing the full rollout of the planned activities, which included community education, improved sanitation facilities, and water purification efforts (described in more detail for each community in Supplemental Table S1). (The survey and community engagement activities—key informant interviews, focus group discussions, and town hall meetings—were applied in all three States. See Section 2.4, below and Table S1).

##### Rationale for Non-Representative Sampling

As this study did not aim to achieve statistical representativeness across the broader population, the selection of sites was not random. Instead, the study sought to capture data from areas where cholera transmission posed significant public health risks. This targeted approach was necessary to assess the effectiveness of the intervention in communities where access was possible, though it limits the generalizability of the findings to the entire country. The selected communities were not intended to represent the full diversity of Nigerian contexts but were strategically chosen to maximize the potential impact of the health education program.

##### Selection of Survey Participants

A randomized approach was used to select individuals for the surveys, with 60 participants from Delta, 70 from Ondo, and 50 from Enugu. Participants were randomly selected from specific areas within 1–2 km of local water sources in each region. This approach ensured that participants were directly involved in water practices that impacted cholera transmission risk.

To determine the appropriate sample size for detecting a significant difference in cholera knowledge and water safety practices between pre- and post-intervention measurements, we conducted a power analysis using the G*Power 3.1 software [20]. The analysis was based on the following assumptions:

- Effect Size (Cohen’s d): A medium effect size (Cohen’s d = 0.5) was assumed for the intervention’s impact on cholera-related knowledge and safe water practices. This effect size is based on previous public health education intervention studies in similar settings, as discussed by Sullivan and Feinn [21].
- Alpha Level (α): The standard significance level was set at 0.05.
- Power (1-β): A power level of 0.80 (80%) was chosen, which is the conventional threshold for detecting meaningful effects with a low risk of Type II errors.
- Test Type: A paired sample *t*-test was used as the study involved comparing pre- and post-test scores within the same individuals (i.e., repeated measures).
- Expected Attrition Rate: The calculation assumed an attrition rate of 10%, assuming that some participants may drop out during the study.

Using these parameters, the power analysis indicated that a minimum sample size of 64 participants would be necessary to detect a medium effect size (Cohen’s d = 0.5) at an alpha level of 0.05 with 80% power.

**Figure 2:**
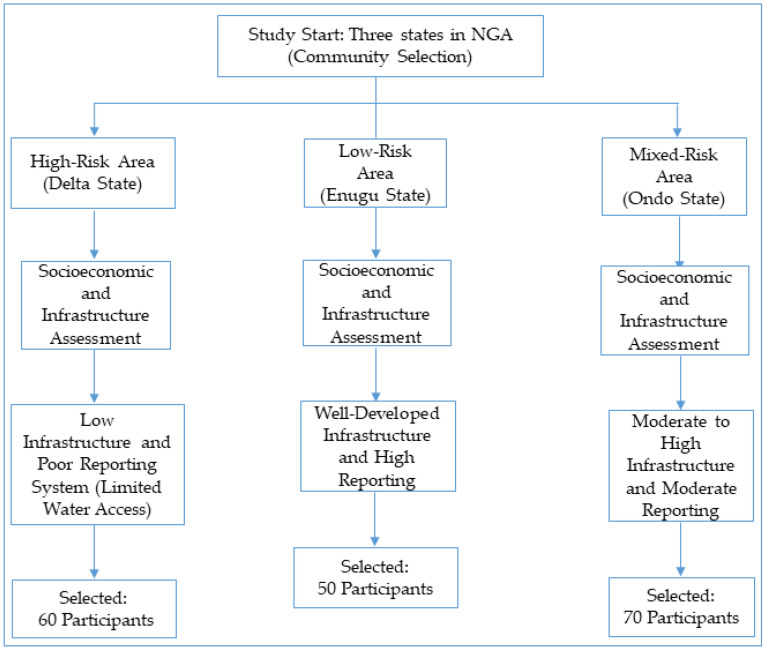
Sampling strategy for community selection and risk categorization in cholera prevention study in rural Nigerian communities.

The following flowchart (Figure 2) illustrates the process of community selection and the risk categorization of the three Nigerian states (Enugu, Delta, and Ondo). The communities were classified based on their water infrastructure, cholera reporting rates, and socioeconomic factors (such as education and income levels). The goal was to examine how these factors influenced cholera transmission and prevention behaviors. This process allows for a stratified sampling approach that considers the varying risk levels of each community to ensure a representative sample across different types of communities.

### 2.2. Timeline of the Study

The research took place over a 12-month period to assess both baseline knowledge and the effect of intervention over time (Table 2).

### 2.3. Educational Interventions and Implementation Strategies

The educational intervention focused on raising awareness of cholera symptoms, transmission, and prevention while also addressing local water stewardship practices, such as the washing of slaughtered animals in water sources. The intervention was designed based on the WHO’s Tailoring Immunization Programmes guidebook [22], the Nigerian Centre for Disease and Control National Public Health Multi-Hazard Emergency Preparedness and Response Plan [23], and the CDC Community-level Interventions framework [24] to ensure relevance, effectiveness, and sustainability. Several components of the intervention were specifically tailored to the local context and community needs, with a focus on behavioral change communication (Table 3).

To reinforce the messages shared during the discussions, health messaging materials such as pamphlets and visual aids were developed and distributed. These materials, written in local languages and featuring simple illustrations, made the key messages easily accessible to participants of all literacy levels. Practical demonstrations, including proper water treatment techniques, were also incorporated into the education sessions. These hands-on activities, especially focused on situations like washing animals or clothes in water sources, helped participants understand how to implement the practices in their daily lives.

A targeted approach ensured that the intervention reached those at risk for cholera infection, with special attention given to women, children, and individuals involved in food preparation and animal husbandry. The program also involved local politicians and village leaders—trusted figures within the community—to encourage participation and reinforce the importance of hygiene education. This community mobilization strategy helped increase attendance at the group discussions and enhanced the credibility of the health messages being delivered. The FGDs and the broader hygiene education efforts worked together to inform the intervention, providing both qualitative and practical insights that allowed the program to be fine-tuned and more effectively address cholera prevention in the communities.

#### 2.3.1. Metrics and Scale for Behavioral Change

To quantify the impact of the intervention, a behavioral change scale was employed, assessing participants’ knowledge, attitudes, and practices (KAP) before and after the educational sessions. The KAP scale, which is widely used in public health research to measure the effectiveness of hygiene and sanitation interventions [25,26], consisted of 20 questions divided into three domains: knowledge of cholera transmission, attitudes toward sanitation practices, and self-reported behavior changes related to water treatment and hygiene practices. The pre-test and post-test design allowed for direct comparison of participants’ responses, quantifying improvements in understanding and changes in behavior. It is important to note that this scale was used to provide a quantitative value for comparative purposes and as a guide for future studies (see Section 4.3). The questionnaire, developed by the Foundation in Nigeria, was exploratory and did not go through a formal public health validation process, nor were reliability coefficients determined [27].

For example, participants were asked to rate their ability to identify cholera symptoms, understand transmission modes, and correctly apply water treatment techniques using a Likert scale (1 = Strongly Disagree to 5 = Strongly Agree). Behavioral changes, such as water treatment, handwashing, and latrine usage, were measured using a scale ranging from 1 = Never to 5 = Always to reflect the frequency of safe hygiene practices. Scores were aggregated to generate a composite index of safe water practices and cholera prevention behaviors, allowing for a clear measure of change across the entire sample.

#### 2.3.2. Quantifying Improvements

Improvements in safe water practices were quantified through a combination of self-reported behavior changes (e.g., increased use of safe water treatment methods) and observed practices during community visits. Participants’ water treatment behaviors were assessed by asking whether they had adopted new practices, such as boiling or chlorinating water, following the educational sessions. The results from the post-test revealed a significant increase in these behaviors: 72% of participants who had previously never treated water reported using treatment methods post-intervention, a notable shift attributed to the hands-on demonstrations during the education sessions.

In addition to water treatment, other key practices, such as handwashing with soap and safe waste disposal, improved. The use of latrines increased by 35% as participants learned about the health risks of open defecation, which was common in the target communities before the intervention. These improvements were statistically significant, as measured by paired *t*-tests comparing pre-and post-test scores, demonstrating the success of the intervention in altering both knowledge and behavior.

#### 2.3.3. Community Engagement and Tailored Interventions

The program’s effectiveness was enhanced by its participatory approach, which involved participants actively engaging in group discussions, practical demonstrations, and local leadership involvement. Village leaders and local politicians, trusted figures in the communities, played a pivotal role in encouraging attendance and reinforcing the health messages. This community mobilization strategy was critical in increasing the program’s reach, fostering trust, and ensuring that participants not only heard but internalized the educational content.

#### 2.3.4. Practical Demonstrations

One of the unique aspects of the intervention was the incorporation of practical demonstrations. For example, during the educational sessions, participants were shown proper techniques for water treatment, such as boiling water and using chlorine tablets. In addition, demonstrations of animal washing practices emphasized the need to avoid using water sources for washing slaughtered animals, a common practice that contributed to water contamination in these areas. The opportunity for hands-on learning reinforced key messages, making them more relatable and easier to implement in participants’ daily routines.

- Water Treatment Practices: Community members were shown step by step how to boil water or apply chlorine and were given the opportunity to practice the techniques themselves under the supervision of the health workers. Participants were also taught how to use locally available materials such as cloth or sand for simple water filtration, which could remove visible debris and reduce the risk of contamination.
- Handwashing Demonstrations: Proper handwashing was a key focus, especially regarding food preparation and caring for young children. Demonstrations highlighted the proper technique (using soap and water, scrubbing for at least 20 s), which was reinforced with group practice.
- Animal Washing: Given the cultural norm of washing slaughtered animals in communal water sources, practical demonstrations illustrated safer practices, such as washing animals in separate, designated areas that would not contaminate water sources. Participants were also shown how to properly dispose of animal waste and clean utensils used for food preparation to reduce contamination risks.

In addition to water treatment, the program addressed sanitation practices that reduce cholera risk. The emphasis was on building and maintaining latrines and the use of designated waste disposal areas. Community health workers facilitated discussions on the importance of proper waste management, particularly in preventing contamination of drinking water sources. They demonstrated how to construct basic but effective pit latrines using local materials and emphasized the importance of handwashing with soap after using the latrine or handling food.

#### 2.3.5. Community-Based Education Through Group Discussions

The intervention was primarily delivered through community-based group discussions led by trained community health workers (CHWs). These interactive and participatory sessions allowed community members to voice their concerns, ask questions, and contribute their knowledge and practices. The discussions were tailored to local cultural practices and the specific cholera risks prevalent in each community. 

Health messaging was reinforced through various materials that complemented the group discussions. Pamphlets, posters, and other visual aids were created using local languages and simple illustrations to ensure accessibility for all literacy levels. These materials emphasized key messages, including the importance of boiling or filtering water, handwashing with soap, and using latrines. The visual aids were particularly helpful in conveying water treatment techniques and hygiene practices that might otherwise be challenging to explain through words alone. These materials were distributed to all participants at the end of each session, allowing them to review the information at their own pace and share it with other community members.

#### 2.3.6. Role of Local Leaders and Community Mobilization

A key aspect of the intervention was the involvement of local leaders, including traditional leaders and village politicians. These individuals, who were trusted and respected within the community, played a critical role in ensuring community engagement and encouraging attendance at the group discussions. Their involvement was operationalized in the following ways:

- Community leaders facilitated participation by endorsing the program and encouraging community members, particularly those from vulnerable groups (e.g., women, children, and food preparers), to attend the discussions.
- Traditional leaders helped in addressing potential cultural barriers, such as resistance to new water treatment practices or sanitation habits. Their involvement helped to bridge the gap between the intervention’s messages and the community’s traditional beliefs and practices.
- Local leaders also participated in the demonstrations to reinforce the health messages further. Their active engagement helped improve community buy-in and trust in the intervention, ensuring that the program was perceived as legitimate and aligned with community values.

#### 2.3.7. Targeted Approach and Adaptation

The hygiene education program was targeted to address the needs of the populations most at risk for cholera, with a particular focus on vulnerable groups such as the following:

- Women, especially those responsible for domestic water collection and food preparation, received detailed education on safe water handling and sanitation practices.
- Children, who are particularly susceptible to cholera, were also a focus. Special attention was given to handwashing techniques and ways to involve children in safe water practices.
- Animal husbandry workers were given additional training on safe practices around the washing of animals and disposal of animal waste to prevent contamination of water sources.

The program also adapted over time based on community feedback and field observations. This flexibility allowed the intervention to be fine-tuned according to each community’s specific challenges and cultural beliefs. For example, in some areas, the practice of washing animals in communal water sources was deeply ingrained. In these cases, the program focused on showing alternatives, such as using separate washing stations or teaching participants how to clean animals using clean water sources rather than community wells.

#### 2.3.8. Evaluation and Adjustments

The evaluation of the hygiene education program was ongoing throughout the intervention. Data from the focus group discussions (FGDs) and surveys were used to assess the program’s effectiveness and inform continuous improvements. The information gathered helped the research team to refine the messaging and delivery methods to better address the barriers identified during community interactions.

### 2.4. Data Collection Tools

This study employed a mixed methods approach to assess the effectiveness of water, sanitation, and hygiene (WaSH) interventions on cholera awareness, prevention behaviors, and incidence. The data collection process included a combination of semi-structured surveys, focus group discussions (FGDs), and key informant interviews. These methods were used to gather both quantitative and qualitative data, offering a comprehensive understanding of how cultural practices, local conditions, and interventions impacted cholera transmission in the three Nigerian states: Delta (high-risk, no intervention), Ondo (mixed-risk, partial intervention), and Enugu (lower-risk, full intervention).

#### 2.4.1. Surveys

The survey questions were designed using closed-ended and open-ended questions based on a review of previous research on cholera outbreaks and cultural practices related to hygiene and sanitation [9,10]. (The full survey is provided in Supplemental Material Table S2).

Examples of Sample Survey Questions:

- How do you believe cholera is transmitted in your community? (Open-ended)
- Do you use any methods to treat your drinking water? (Yes/No)
- Have you ever washed slaughtered animals in water used for drinking or bathing? (Yes/No)
- Do you believe that open defecation contributes to cholera outbreaks? (Yes/No)
- How often do you use sanitation facilities such as toilets or latrines? (Never/Rarely/Often/Always)

The questionnaire was translated into three local languages (Delta Igbo, Yoruba, and Igbo) to ensure accessibility and understanding among participants from different linguistic backgrounds. These translations were critical for ensuring cultural relevance and maximizing the accuracy of responses.

#### 2.4.2. Key Informant Interviews and Town Hall Meetings

In addition to the survey data, key informant interviews and town hall meetings were conducted with community leaders, local health workers, WaSH program coordinators, and community members. These interviews provided a deeper understanding of local sanitation practices, the history of cholera outbreaks, and the challenges faced by the community in cholera prevention efforts. The qualitative data gathered from these sources, including focus group discussions and informal conversations, shed light on several key barriers to effective cholera prevention.

### 2.5. Methodology for Assessing the Cultural Practice of Washing Slaughtered Animals in Drinking Water Sources

To specifically assess the role of washing slaughtered animals, a set of survey questions was designed to understand community behaviors and attitudes toward this practice (Supplemental Table S2). Additionally, the focus group and key informant interviews explored whether alternative water sources were available for this activity and whether community members would be willing to adopt safer practices if educated about the risks. The data were analyzed using thematic analysis for qualitative data, focusing on recurring themes about the cultural significance of these practices and how they interact with waterborne diseases like cholera. For quantitative data, chi-square tests were used to assess whether there was an association between the practice of washing animals in drinking water sources and increased cholera knowledge or cholera incidence in the community.

### 2.6. Data Analysis

To evaluate the effectiveness of the hygiene education program, the study employed paired *t*-tests, chi-square tests, and logistic regression to assess changes in knowledge, attitudes, and behaviors between the pre- and post-test periods. Chi-square tests were used to compare categorical variables, such as participants’ knowledge of cholera symptoms and transmission methods, while paired *t*-tests assessed continuous variables like changes in self-reported behavior frequency. Logistic regression was used to control for socio-economic factors (e.g., income and education) that may have influenced the outcomes. All analyses were performed using R Statistical Software (v4.1.2; R Core Team 2021 [28]).

Data from the surveys were coded and analyzed using both descriptive and inferential statistics. Descriptive statistics (e.g., frequencies and percentages) were used to summarize participants’ demographic characteristics, while chi-square tests were applied to identify associations between cultural practices (e.g., open defecation and animal washing in water sources) and perceptions about cholera transmission. The chi-square test is an effective tool for identifying patterns in categorical data and was selected based on its ability to assess the relationship between traditional practices and cholera knowledge in this context [29]. In addition, regression analysis was performed to explore the relationship between community-level interventions (e.g., WaSH programs) and changes in cholera incidence. Qualitative data from thematic analysis were used to identify common themes and patterns in community beliefs, practices, and responses to health interventions [30].

### 2.7. Ethical Considerations

Ethical approval for the study was obtained from the Nigerian Institutional Review Board (IRB) for the Allen Foundation’s survey with the approval of the Minister of Public Health and collaboration with local health authorities (ethical approval # NHREC-CHOL-2021-002). Oral informed consent was obtained from all participants before data collection. This process included a detailed explanation of the study’s objectives, the voluntary nature of participation, and assurances of confidentiality. Participants were informed that they could withdraw from the study at any point without any consequences. All survey and interview responses were anonymized to protect participant privacy. Data were stored securely, and any personally identifiable information was removed to ensure confidentiality. Given that the study involved young adult participants, special attention was paid to obtaining voluntary consent from their parents. For adult participants, particularly those in rural areas who may not have been familiar with research procedures, volunteers stood with them to explain and discuss the questionnaire processes, ensuring comprehension and addressing concerns [13].

### 2.8. Recruitment and Inclusion Criteria

The inclusion criteria for the study were designed to ensure the representation of relevant community members from cholera-prone areas in Nigeria. Participants had to be residents of one of the selected cholera-prone communities—Ogwashu (Delta), Ikare (Ondo), or Ugbwaka (Enugu)—during the study period. In addition, participants were required to be at least 13 years old to ensure they could provide informed consent and participate meaningfully in the study. This age requirement is also aligned with ethical considerations regarding consent for minors.

Individuals who voluntarily agreed to participate in the study were included to ensure a diverse and comprehensive sample. This allowed for the collection of data from a range of participants, reflecting different genders, ages, and socio-economic backgrounds. The study aimed to capture a variety of perspectives on cholera transmission and prevention, providing a holistic view of local attitudes and practices.

The exclusion criteria were equally important to ensure the integrity of the study. Individuals who did not reside in the designated study areas during the study period were excluded. This ensured that the data accurately reflected the experiences and perceptions of people living in the high-risk communities under investigation. Participants under 13 were excluded due to the ethical challenges in obtaining informed consent from minors. Finally, individuals with cognitive or hearing impairments that would hinder participation in surveys or focus group discussions were excluded as these impairments could affect their ability to provide comprehensive responses, thus compromising the quality of the data.

## 3. Results

The study assessed the effectiveness of a culturally tailored hygiene education intervention aimed at reducing cholera transmission and promoting safe water practices across three rural Nigerian communities. The selected communities included both mixed-risk (Ondo) and low-risk (Enugu) areas based on prior cholera incidence data, as well as consultations with local health authorities and the Allen Foundation. Both sets of communities received the intervention, but differences in cholera incidence and exposure to the intervention were carefully considered in the analysis of results. The intervention’s impact was evaluated using pre-and post-test assessments, with statistical significance explicitly reported. The third community in the high-risk Delta State was also included in the analysis, although the intervention could not be implemented. However, metrics also improved in this State, due to changes in local practices promoted by public health officials which increased awareness, influenced in part by the data collection tools in this study that were implemented (surveys, FGDs, key informant interviews, and Town Halls).

A key outcome of the study was the increase in the number of individuals able to correctly identify cholera symptoms and the number of cases reported to health authorities. This was measured as part of the post-test survey and corroborated through follow-up interviews.

### 3.1. Intervention Impact on Knowledge and Behavior

The results showed statistically significant improvements in participants’ knowledge of cholera transmission and their adoption of safe water practices after the intervention. In particular, the proportion of respondents correctly identifying cholera symptoms (vomiting and diarrhea) and transmission routes (contaminated water and food) increased significantly from the pre-test to the post-test (*p* < 0.001). For example, 80% of participants could identify cholera symptoms in the post-test, compared to 52% in the pre-test (Table 4), illustrating the effectiveness of the educational intervention and community stewardship.

Participants reported increased engagement in safe water practices, particularly water treatment methods. In the pre-test, only 42% of participants treated their water, whereas, post-intervention, this figure rose to 72%. The increase was statistically significant (*p* < 0.001) (Table 5).

The use of latrines showed a significant improvement, particularly in high-risk communities like Ikare and Ogwashi-Ukwu, where open defecation rates were high pre-intervention. The use of latrines increased by 35% in the post-test (*p* = 0.02). The intervention was particularly successful among individuals with higher education levels (secondary school and above) and those from higher income brackets, who were more likely to adopt latrine use.

Cultural Practices Impact: One of the most notable findings from the intervention was the reduction in risky cultural practices, such as washing slaughtered animals in rivers. Pre-intervention, 30% of participants reported engaging in this practice, but this dropped to 15% in the post-test, a statistically significant reduction (*p* = 0.01). This change was particularly pronounced in younger participants (aged 14–24) and those with primary education.

Socioeconomic factors, including education and income levels, influenced the effectiveness of the intervention. Participants with secondary school education and those from higher income brackets showed greater improvements in safe water practices and were more likely to identify cholera symptoms and transmission methods. Conversely, participants with no formal education and those from lower income brackets showed smaller improvements, suggesting the need for more tailored educational strategies for these groups (Table 6).

### 3.2. Demographic Characteristics of Respondents

Table 7 presents the demographic characteristics of the study participants, which include gender, age, educational background, and income level. These socio-economic factors provide crucial context for understanding how knowledge and practices regarding cholera prevention may vary within the population.

#### 3.2.1. Gender Distribution

The study had a fairly balanced gender distribution, with 53% male participants and 47% female participants. Gender did not appear to significantly influence cholera awareness or intervention practices, though gender-based roles in water collection and hygiene could play a more significant role in community-level interventions, especially in higher-risk areas.

#### 3.2.2. Age Distribution

The majority of participants were between 18 and 35 years old (65% combined), representing the primary age group likely responsible for food preparation and water collection in rural settings. This age group also tends to be more engaged in health education and behavioral change programs. The younger group (14–24 years) demonstrated higher engagement in water treatment and cholera knowledge, which may reflect a greater openness to adopting new health practices.

#### 3.2.3. Educational Background

Educational level emerged as a critical factor influencing knowledge and behavior regarding cholera. Of the participants, 14% had no formal education, while 42% had attended primary school, and 44% had secondary school education. Higher education levels were strongly correlated with better knowledge of cholera symptoms and prevention strategies. Participants with secondary education or higher showed a higher tendency to report safer water treatment practices. This highlights the need for tailored educational campaigns that address the gaps in knowledge, especially among those with lower education levels.

#### 3.2.4. Income Level

Income also played a significant role in shaping access to safer water sources and willingness to adopt safer water practices. A large proportion of participants (35%) reported earning between N500 and N1000 per annum, indicating that a significant portion of the population lives in poverty. Individuals with lower income levels had less access to safe water sources and were less likely to afford water treatment options. Furthermore, those with higher income levels (N15,001–N30,000 per annum) exhibited greater awareness and were more likely to engage in safer water practices, likely due to better access to resources, including proper sanitation facilities and treated water.

Overall, these socio-economic factors—especially education and income—played an essential role in shaping participants’ awareness and practices regarding cholera prevention. The data suggest that higher levels of education and income correlate with greater awareness and willingness to adopt safer water treatment practices. Future interventions should consider these socio-economic disparities and target educational efforts to those with lower education levels or limited financial resources to ensure more widespread adoption of cholera prevention measures. Additionally, a comprehensive approach that integrates education with tangible infrastructure improvements, especially in lower-income communities, is critical for reducing cholera incidence in high-risk regions.

### 3.3. Post-Intervention Analysis and Group Comparison

Table 8 presents a summary of the demographic and intervention-specific data, highlighting the changes in participants’ knowledge and practices before and after the interventions and the differences between participants who received the intervention and those who did not.

### 3.4. Changes in Knowledge and Practices

The intervention had varying effects depending on the region and the extent to which participants were exposed to the WaSH programs. Participants in Enugu, where the intervention was fully implemented, demonstrated improvements in both knowledge and practice. Pre-test awareness of cholera transmission was 60%, which increased to 82% after the intervention. Similarly, safe water practices increased from 55% to 80%. This reflects the effectiveness of WaSH programs in enhancing cholera-related knowledge and behaviors in communities with strong intervention exposure.

In contrast, Delta, where no intervention had been implemented, showed more modest improvements. Pre-test awareness was 48%, and post-test awareness only increased to 60%. Safe water practices also improved less, from 35% to 52%. This indicates that changes in knowledge and behavior were limited without intervention, emphasizing the necessity of interventions to drive substantial improvements in cholera prevention.

Participants in Ondo, where partial interventions were implemented, exhibited intermediate changes. Pre-test cholera transmission awareness increased from 50% to 68%, and safe water practices rose from 42% to 60%. This group represents a transitional level of exposure, with some benefits from the intervention but without the full implementation that was seen in Enugu.

### 3.5. Cholera Incidence: Comparing Pre-Intervention and Post-Intervention Rates

**Figure 3:**
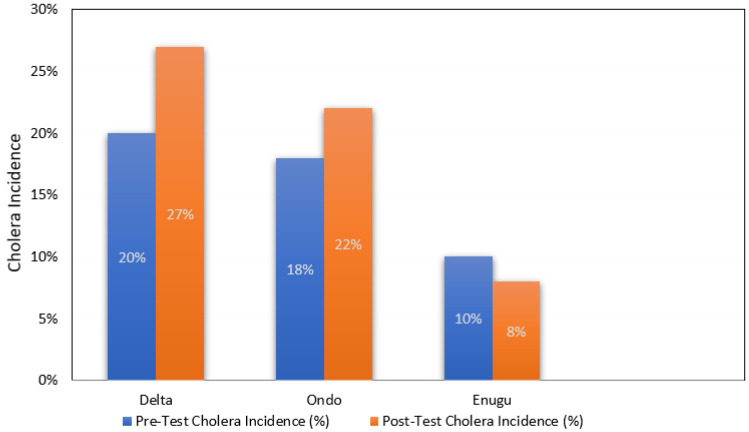
Trends in cholera incidence before and after the intervention in three Nigerian states (data taken from Table 8, which provides 95% confidence intervals (*p* < 0.05)).

Cholera incidence rates followed a similar pattern to the changes in knowledge and practices (Figure 3). In Enugu, where comprehensive WaSH interventions were deployed, cholera incidence decreased from 10% pre-intervention to 8% post-intervention. The minimal reduction in cholera cases might be attributed to the lower baseline incidence in this area, but it still suggests that sustained education and access to safe water sources can effectively reduce cholera outbreaks.

For Delta, which had the highest pre-test cholera incidence (20%), the post-test rate increased to 27%. This could be explained by the lack of a comprehensive intervention in this area, where participants continued to engage in high-risk behaviors, such as washing slaughtered animals in drinking water sources. Without consistent exposure to WaSH programs, behavior change was not significant, leading to a higher rate of cholera incidence. At the same time, improved reporting of cholera as a result of the intervention could show up as increased incidence, as potentially in Ondo.

In Ondo, cholera incidence was increased from 18% to 22%, suggesting that partial interventions may only have been effective in increasing reporting. The presence of WaSH programs in some areas likely helped reduce the incidence, but the uneven coverage and exposure to the program may have diminished its overall impact.

### 3.6. Statistical Analysis

#### 3.6.1. Cross-Tabulation Results

Table 9a–d present the cross-tabulation results for the relationships between cholera incidence and the practice of open defecation and the practice of washing slaughtered animals in source waters and hygiene education (Table 9a–c). Table 9d shows the relationship between the use of chlorine and participants’ knowledge of cholera transmission.

These findings indicate a significant relationship between cholera incidence and the practice of washing slaughtered animals in water sources. No other cross-tabulation results were significant.

#### 3.6.2. Logistic Regression Analysis Results of Key Behavioral and Knowledge Factors Influencing Cholera Incidence

Table 10 evaluated factors influencing cholera incidence. WaSH program awareness was significantly associated with reduced cholera incidence (OR = 3.91, *p* = 0.005), highlighting the importance of water and sanitation education in preventing cholera. Specifically, it is important provide as much information to communities as possible on WaSH programs—through all culturally acceptable forms of communication. In contrast, other factors such as access to healthcare resources, open defecation, and chlorine use for water treatment did not show statistical significance, suggesting that awareness and education may have a more direct impact on cholera prevention. The odds ratios (ORs) indicate the strength and direction of the association, while the *p*-values provide insight into the statistical significance of each factor. An OR greater than 1 suggests a higher likelihood of cholera incidence, whereas an OR less than 1 indicates a lower likelihood. Factors with *p*-values greater than 0.05 are not statistically significant. It is important to note that potential collinearity issues may arise if variables like WaSH awareness, healthcare access, and open defecation are highly correlated. This can lead to unstable coefficient estimates and inflated standard errors, making it difficult to assess the unique impact of each factor on cholera incidence.

### 3.7. Qualitative Findings from Community Engagement, Including Key Informant Interviews, Focus Group Discussions, and Town Hall Meetings

Qualitative findings from community engagement activities, including key informant interviews, focus group discussions, and town hall meetings, are summarized in Table 11. The findings highlight key cultural practices, perceptions of water safety, and barriers to cholera prevention that were identified through direct conversations with community members and local health officials.

Many community members did not see the practice of washing slaughtered animals in water sources as a major risk for cholera transmission. In fact, some participants indicated that this was a longstanding tradition and had not been associated with illness in their community. Similarly, there was a general trust in local water sources, with many participants stating that they believed the water was “clean enough” despite visible contamination or potential risk factors. Furthermore, town hall meetings provided insights into the logistical and infrastructural challenges that hinder cholera prevention. Many participants cited the lack of proper sanitation, limited access to clean water, and inadequate hygiene education as significant barriers to improving sanitation practices. These insights were important in understanding the gap between knowledge and practice in cholera prevention and the social and cultural context within which these practices occur.

## 4. Discussion

### 4.1. Cultural Context

One of the most important findings from the study was the identification of cultural practices, particularly the washing of slaughtered animals in drinking water sources, as a significant risk factor for cholera transmission (*p* = 0.019). This practice was especially prevalent in rural communities with limited access to clean water and sanitation facilities, underscoring the role of water contamination in cholera outbreaks [31,32]. Although there is an increasing literature on contamination of water through slaughterhouse practices, including in Nigeria [33,34], there is little information on this culturally established practice at the community level. Previous studies have focused on the importance of WaSH in reducing cholera, e.g., [35,36,37], without emphasizing the need to address cultural practices such as washing slaughtered animals.

The results from this study underscore the complex interplay of socio-cultural practices, local infrastructure challenges, and community resistance to change in addressing cholera transmission. Cholera prevention, especially in rural Nigerian settings, is deeply rooted in cultural norms and traditional beliefs, many of which complicate the adoption of modern health practices [38,39,40]. These cultural dynamics and infrastructural barriers are often related, creating a multi-layered challenge for health interventions [41,42]. Drawing upon qualitative data, this section explores the socio-cultural context that hinders behavior change, the role of traditional beliefs and practices, and the infrastructural gaps that exacerbate cholera outbreaks, with a particular focus on the insights derived from the focus group discussions (FGDs).

A significant barrier to cholera prevention identified through the FGDs was community resistance to adopting modern hygiene and sanitation practices. In many areas, particularly in Ondo State, long-standing cultural practices were so deeply ingrained that they often took precedence over health education efforts. For example, the washing of slaughtered animals in communal water sources mentioned above was a widespread practice, and many participants did not perceive it as a health risk. This is consistent with findings in other studies in Nigeria [6,43], which note that cultural practices are often not only common but are viewed as normal or even sacred. These practices are often resistant to change because they are embedded in cultural identity and are passed down through generations [44,45].

The resistance is further compounded by the trust in traditional knowledge systems over modern scientific approaches. Traditional healers, for example, continue to hold significant influence in rural Nigerian communities, and their practices—ranging from herbal remedies to spiritual cleansing—are often favored over medical advice [46]. Traditional healers may even discourage the use of modern sanitation practices, including the use of latrines or chlorinated water, which they may view as unnecessary or counterproductive to their healing practices. This poses a direct challenge to the promotion of practices such as boiling water, using latrines, and the consistent application of hygiene interventions.

The influence of traditional beliefs is not limited to medical practices; it also extends to water management practices. For instance, in Ondo, many community members continued to trust their local water sources despite evidence of contamination, viewing these sources as pure or blessed due to their historical or spiritual significance. In such communities, behavioral change interventions must be more than just education—they must acknowledge and integrate the cultural significance of these practices, thereby crafting interventions that respect local customs while promoting safer practices [47,48,49,50,51,52].

Beyond cultural resistance, inadequate sanitation infrastructure remains one of the most pressing issues in cholera prevention [53,54]. As noted in the FGDs, there were significant gaps in sanitation facilities, particularly in rural communities like those in Ondo. The lack of access to clean water and adequate waste disposal systems is a critical factor contributing to the persistence of cholera outbreaks. In areas where latrines are either non-existent or poorly maintained, people often resort to open defecation, which increases the likelihood of water source contamination and the spread of disease [44,45]. Here there may be an opportunity to explore community-based approaches where local healers and leaders are trained in basic hygiene and water management techniques. This allows them to serve as local advocates, bridging the gap between modern health practices and traditional beliefs. Such initiatives can build trust and make health interventions more socially acceptable, increasing their effectiveness and long-term sustainability [32,45].

Despite some progress in sanitation through WaSH programs, the rural–urban divide in infrastructure remains stark. As seen in Ondo State, the distance between water sources and sanitation facilities often forces families to rely on unsafe water for drinking and cooking. This was further exacerbated by seasonal changes that often render water sources even more susceptible to contamination, especially during the rainy season when floods wash waste into previously clean water sources. Without government investment in proper waste management systems or sewage treatment, these communities remain at a high risk of cholera outbreaks [53].

Participants in the FGDs from high-risk regions emphasized that the lack of infrastructure goes hand in hand with the absence of local government support. While local leaders may advocate for better infrastructure, there is often a disconnect between political promises and actual implementation due to a lack of resources and political will. The shortage of funds allocated for water treatment and sanitation projects has further entrenched this issue, making it difficult for communities to move beyond their reliance on unsafe water sources. The involvement of local leaders, as seen in the study, was critical to increasing community participation in the hygiene education sessions. However, local governance remains weak in many regions, especially in rural and marginalized areas. In Ondo, while village leaders played a role in promoting hygiene education, they also expressed frustration with the lack of support from higher levels of government. These leaders are often inundated with responsibilities that go beyond health issues, such as political and economic challenges. This lack of coordination and inter-sectoral collaboration limits the effectiveness of cholera prevention programs as local governance structures are unable to adequately address both health and infrastructure needs.

### 4.2. Behavioral Change

The quantification of behavioral change was based on a combination of self-reported surveys to the health department and observational data. Participants were asked to report on their water treatment practices, handwashing habits, and the use of latrines before and after the intervention. This allowed for a clear measure of changes in knowledge and behavior related to cholera prevention. Self-reports were crucial in evaluating the extent to which participants had internalized the health messages and made real-life changes. A critical aspect of the behavioral change measured was stewardship of water resources. In high-risk communities, the intervention aimed at not only improving individual behaviors but also at fostering community-level accountability in managing water sources. In particular, the mismanagement of water resources such as washing animals in rivers. As a result, increased community stewardship was observed as participants became more aware of the consequences of their actions on water quality and cholera transmission. One of the key findings of the intervention was the significant improvement in water treatment practices. In high-risk communities, participants reported higher levels of boiling or chlorinating their drinking water post-intervention compared to the pre-intervention period. This behavior change was particularly prominent among participants who received the full intervention, where practical demonstrations of water purification techniques helped reinforce the importance of these measures. In lower-risk areas, although water treatment practices improved, the changes were less significant, reflecting the less intensive nature of the intervention.

Knowledge about cholera symptoms and the importance of timely reporting also showed notable improvements. In high-risk areas, participants were better able to identify symptoms such as diarrhea, vomiting, fever, and dehydration. However, knowledge gaps remained, especially in communities with less extensive interventions. Misunderstanding of cholera transmission and a lack of trust in water infrastructure, combined with the underreporting of cases, remained a challenge despite the improvements in overall knowledge.

The analysis of WaSH program awareness revealed a significant relationship between knowledge of WaSH programs and reduced cholera incidence (OR = 3.91, *p* = 0.005). Participants who had been exposed to WaSH interventions were more likely to adopt safer water practices, such as water treatment, and report improved health-seeking behaviors. WaSH programs have been shown to effectively raise awareness of cholera transmission risks and encourage behaviors that reduce cholera incidence [32,35,55,56]. However, in this study, the success of these programs varied across communities, particularly between those that had already been exposed to interventions and those that had not. For example, in communities where WaSH programs had been implemented (Enugu), participants showed significantly higher levels of awareness and were more likely to engage in cholera-preventive behaviors. Conversely, communities that had not been exposed to WaSH programs (Delta) continued to engage in high-risk behaviors, such as washing slaughtered animals in drinking water sources. This underscores the importance of expanding WaSH interventions to underserved areas to ensure that all communities benefit from the education and resources needed to prevent cholera outbreaks [8,35,57].

The lack of statistical significance found in the logistic regression results (OR = 0.70, *p* = 0.457) between hygiene education and cholera awareness was not anticipated. While educational interventions improved cholera knowledge and safe water practices, these did not translate into a measurable reduction in cholera cases. There are a number of confounding factors, which include socio-cultural beliefs, and deep-seated mistrust of health infrastructure, discussed above. However, there may also be the fact that cholera awareness resulted in improved reporting of numbers of cases, resulting in the appearance of an increase in cholera incidence.

A critical theme that emerged was the role of local governance in overcoming these barriers. While there was general awareness of cholera risks among community leaders, limited resources and political will often hinder the implementation of effective sanitation and water treatment interventions [36,58]. Strengthening local governance and improving inter-sectoral coordination were seen as critical steps in addressing these challenges and reducing cholera incidence in these regions.

### 4.3. Limitations and Future Directions

This study provides valuable insights into the role of cultural practices and beliefs in cholera transmission. However, it is important to acknowledge several limitations that affect the generalizability of the findings. First, the research was conducted in three states of Nigeria—Enugu, Delta, and Ondo—each with distinct socio-economic conditions, water quality, and sanitation infrastructure. Therefore, the findings may not be broadly applicable to other regions of Nigeria, particularly northern parts of the country, where security concerns limited data collection, and water and sanitation infrastructure may be vastly different. Regional variations in water quality and infrastructure can significantly impact the effectiveness of health interventions and complicate the interpretation of results on a national scale. For example, Enugu, which is categorized as a lower-risk state, has relatively better water quality and infrastructure compared to Delta and Ondo, both of which are higher-risk areas with poorer sanitation and greater reliance on unsafe water sources. These infrastructural differences might contribute to the disparity in cholera incidence across the states. In Enugu, participants may have had fewer barriers to practicing safe water treatments. In contrast, Delta and Ondo face persistent water contamination, inadequate sanitation, and higher rates of open defecation, which exacerbate the risk of cholera outbreaks. Such differences in infrastructure suggest that the findings may not be applicable to regions with poorer water quality or less robust sanitation infrastructure. Therefore, the generalizability of the findings to other parts of Nigeria or similar countries with different water and sanitation conditions remains limited. There are also other variables that can influence cholera that will vary between regions, such as seasonal variability, climate, and the large variety of point and non-point sources of contamination [59] not considered in this study.

In addition to infrastructure, socio-economic conditions also vary between the study sites. Communities in Delta and Ondo generally face higher poverty levels and lower educational attainment than Enugu. These socio-economic factors likely influence water treatment practices and engagement with health interventions as those with lower income levels and limited education may have less access to resources like safe water sources or sanitary facilities, even if they understand the health risks. Socioeconomic disparities—such as differences in income, education, and access to resources—pose significant barriers to the adoption of safe water practices and, by extension, the effectiveness of educational interventions.

Moreover, the study’s reliance on focus group discussions and surveys limits the depth of data collected, particularly in assessing long-term changes in cholera incidence. Sustainability of these interventions is, therefore, in question and should be addressed through longer-term studies. A six-month period is a short time frame to assess whether interventions can be successful over the longer term. Conversely, six months may not be long enough to detect changes in behavioral patterns. Longitudinal studies that track changes over time, including the persistence of cholera outbreaks and the adoption of safe water practices, would provide more comprehensive insights into the long-term impact of health interventions [60,61,62]. Although the Allen Foundation randomly selected all participants and there should be no selection bias at the individual level, the quasi-experimental design did lack a control group. This may limit the ability to draw definitive causal conclusions about the intervention’s effectiveness. The absence of a control group can threaten internal validity as it becomes difficult to rule out other factors influencing the observed outcomes. Future randomized controlled trials (RCTs) would be valuable in strengthening causal claims by providing more robust evidence of the effectiveness of interventions. Unlike observational studies, RCTs can minimize biases and confounding variables by randomly assigning participants to treatment and control groups, ensuring that any observed effects on cholera incidence can be more confidently attributed to the WaSH program rather than other factors. This would provide a clearer insight into the direct impact of water, sanitation, and hygiene interventions on reducing cholera incidence.

Future research should explore sustainable, long-term intervention strategies, such as integrating WaSH programs into school curricula, leveraging mobile health technologies for continuous education, and establishing community-led monitoring systems to ensure sustained behavior change. In addition, the geographic scope should be expanded to include a more diverse range of communities across Nigeria, particularly in areas where cholera is endemic and where water and sanitation infrastructure are most inadequate. Lastly, while the study demonstrated improvements in knowledge and safe water practices, these changes did not translate into significant reductions in cholera incidence, particularly in high-risk regions like Delta and Ondo. The increases in cholera cases (Delta: from 20% to 27%; Ondo: from 18% to 22%) underscore a critical point: education alone is insufficient to prevent cholera outbreaks. The cultural context—where harmful practices such as washing slaughtered animals in water sources and open defecation remain entrenched—represents a formidable barrier to the effectiveness of education programs. Participants in the study were often reluctant to abandon these practices, even when made aware of the associated health risks. This finding highlights the need for culturally sensitive interventions that go beyond basic education to address deeply ingrained cultural norms and behavioral change. Additionally, inadequate sanitation infrastructure was a significant challenge, with many communities lacking the resources to implement safe water and hygiene practices despite understanding the risks. This underscores the need for a dual approach to cholera prevention—one that combines health education with investments in water and sanitation infrastructure. Local governments, NGOs, and international organizations must prioritize improving sanitation facilities in high-risk regions, especially in states like Delta, where poor water quality continues to exacerbate cholera outbreaks.

## 5. Conclusions

This study provides compelling evidence on some of the factors driving cholera transmission in Nigeria and offers actionable recommendations for reducing its incidence. By examining the impact of WaSH programs, cultural practices, and community engagement, we uncover both the challenges and opportunities in cholera prevention. The study highlights several key findings that demonstrate the effectiveness of culturally tailored, multifaceted interventions in reducing cholera outbreaks. Both the qualitative findings and the quantitative data provide a holistic view of the challenges that communities face in preventing cholera. These insights will be crucial for designing targeted, community-driven health interventions that address both the cultural and infrastructural factors contributing to cholera transmission.

Multi-pronged, culturally sensitive interventions are Nigeria’s most effective strategy for cholera prevention. While education on waterborne diseases and hygiene practices is crucial, it must be paired with infrastructure improvements, behavioral interventions, and community-driven strategies that respect local customs. To reduce cholera incidence, future interventions must focus on the following:

1. Enhanced WaSH education to improve cholera knowledge and encourage safer water practices.
2. Targeted behavioral change programs that address cultural practices and perceptions surrounding hygiene and water management.
3. Improved sanitation infrastructure to ensure the availability of clean water and proper waste disposal.
4. Community engagement and local leadership to create culturally appropriate, sustainable solutions.

By combining these approaches, cholera prevention efforts in Nigeria can be more effective and sustainable, ultimately reducing the burden of cholera and improving public health outcomes.

## Figures and Tables

**Table 1:** Characteristics of the three communities.

Ogwashu (Delta > 5 Cases per 1000 People Annually)	
High Risk	Chosen due to its recurrent cholera outbreaks, characterized by high rates of infection and frequent cholera episodes reported in recent years. This community’s inclusion allowed for a focus on areas facing more persistent cholera challenges.
Ikare (Ondo < 5 cases per 1000 people annually)	
Mixed Risk	Represents an area with lower cholera outbreaks, selected to explore how cholera prevention efforts work in areas with less frequent but still notable cholera cases. Ikare’s selection reflected a region with periodic outbreaks rather than the high recurrence seen in Delta.
Ugbwaka (Enugu < 5 cases per 1000 people annually)	
Low Risk	Chosen due to its historically lower cholera incidence. This community’s inclusion helped balance the study, providing insights into how cholera prevention efforts function in a setting where cholera is a less-frequent concern.

**Table 2:** Timeline.

Date	Activity
30 November 2021	Initial meetings with public health officials and the Allen Foundation to discuss the cholera outbreak and finalize survey designs.
1 December 2021–1 January 2022	Pre-test surveys were administered to 180 participants across the three communities to assess baseline knowledge of cholera and water management.
Jan 2022–February 2022	Data analysis and planning for interventions, including design and logistics for educational seminars and community engagement.
March 2022	Educational interventions delivered through community seminars and public health rallies. These interventions were based on a tailored educational model, focusing on the following: Cholera Transmission: How cholera spreads through contaminated water and improper sanitation. Preventive Measures: How to treat drinking water, the importance of sanitation, and proper waste disposal. Recognition of Cholera Symptoms: Diarrhea, vomiting, dehydration, and the critical need for early treatment. Actionable Steps: How to report cases and seek immediate medical attention. These sessions were designed with both informative lectures and interactive elements, including role-playing activities, group discussions, and Q&A sessions to enhance participant involvement.
March 2022–August 2022	A six-month waiting period allowed the research team to observe natural changes in cholera-related behaviors, including water stewardship practices and increased reporting of cholera symptoms, as well as to track any increases in community engagement following the seminars.
September 2022	Post-test surveys were administered to the same 180 participants, measuring improvements in cholera knowledge, water treatment behaviors, and reporting practices.
October–November 2022	Post-test data were analyzed to evaluate the effectiveness of the educational interventions and compare the findings to pre-test data.
December 2022	Results and reports were shared with local health authorities and relevant stakeholders for future action planning.

**Table 3:** Educational intervention.

Duration and Frequency of Sessions	A total of three sessions were held over the course of one week per community, each lasting 1–2 h. These sessions were designed to be intensive but manageable, with breaks for group interaction and feedback. To ensure accessibility for all participants, the sessions were held in central community locations such as schools, local meeting halls, and health clinics.
Content Covered	Session 1: Introduction to cholera, modes of transmission, and common misconceptions (e.g., the belief that cholera is only spread by human-to-human contact). Session 2: The importance of safe water practices (e.g., treating water and using sanitation facilities) and the risks of washing slaughtered animals in water sources. Session 3: Recognition of cholera symptoms (vomiting, diarrhea, and dehydration), effective dehydration management (ORS), and how to report suspected cases to local health officials.
Frequency of Messages	The key messages—such as cholera transmission, prevention practices, and the importance of reporting symptoms—were reinforced during each session. In addition, messages were disseminated in public health rallies organized in each community, reaching a broader audience who were not part of the formal seminar sessions. Post-session follow-up involved distributing printed materials (flyers and posters) summarizing key points and SMS-based reminders to participants, particularly targeting mobile phone users in each community.
Participant Involvement	To foster active participation, the seminars included interactive group activities, such as small-group discussions on local cholera myths and the creation of community action plans for water stewardship. Participants were encouraged to identify key community leaders (e.g., village chiefs and health workers) who could act as champions of change, reinforcing the messages and encouraging others to adopt safer water practices.
Community Partnerships	The Allen Foundation played a critical role in community mobilization and logistical support, including organizing seminar venues, recruiting local facilitators, and distributing educational materials. Local health authorities and community leaders were actively involved in facilitating sessions, ensuring that the content was culturally appropriate and that information was accurately delivered.

**Table 4:** Knowledge of cholera symptoms pre- and post-test (significant results in bold).

Knowledge Item	Pre-Test	Post-Test	t-Value	*p*-Value
Correct identification of symptoms	52%	80%	7.5 (179)	**<0.001**
Correct transmission routes	58%	85%	7.3 (179)	**<0.001**

**Table 5:** Behavioral changes in water treatment practices pre- and post-test (significant results in bold).

Practice	Pre-Test	Post-Test	t-Value	*p*-Value
Treating water	42%	72%	8.2 (179)	**<0.001**
Handwashing with soap	55%	78%	6.2 (179)	**<0.001**
Safe disposal of waste	50%	76%	7.0 (179)	**<0.001**

**Table 6:** Socioeconomic impact on safe water practices post-intervention (significant results in bold).

Socio-Economic Variable	Safe Water Practices Post-Test	*p*-Value
No education	50%	**<0.05**
Primary school level	68%	**<0.05**
Secondary school level and above	85%	**<0.001**
N500–N1000 income bracket	60%	**<0.05**
N15,001–N25,000 income bracket	80%	**<0.001**

**Table 7:** Demographic variables frequency table (additional details are provided in Supplemental Table S3).

Characteristics	Frequency (%)
**Gender Distribution**	
Female	85 (47%)
Male	95 (53%)
**Age Distribution**	
14–17 years	22 (12%)
18–24 years	43 (24%)
25–35 years	73 (41%)
35–44 years	12 (7%)
45 years and above	30 (17%)
**Educational Background**	
No education	29 (16%)
Primary school level	76 (42%)
Secondary school level	75 (42%)
Income Level *	
N500–N1000 per annum	60 (33%)
N1001–N5000 per annum	36 (20%)
N5001–N15,000 per annum	20 (11%)
N15,001–N25,000 per annum	34 (19%)
N25,000–N30,000 per annum	30 (17%)

* 1000 Nigerian Naira is equivalent to USD 0.67 (accessed on 2 March 2025).

**Table 8:** Summary of participants and pre/post-intervention changes (95% confidence intervals in parentheses).

State	Risk Level	# of Participants	Intervention Exposure	Pre-Test Awareness (Cholera Transmission)	Post-Test Awareness (Cholera Transmission)	Pre-Test Safe Water Practices (%)	Post-Test Safe Water Practices (%)	Cholera Incidence Pre-Test (%)	Cholera Incidence Post-Test (%)
Delta	High Risk	60	No Intervention	48% (42.3, 53.7)	60% (54.3, 65.7)	35% (28.8, 41.2)	52% (45.2, 58.8)	20% (14.2, 25.8)	27% (21.3, 32.7)
Ondo	Mixed Risk	70	Partial Intervention	50% (44.1, 55.9)	68% (62.1, 73.9)	42% (35.3, 48.7)	60% (53.4, 66.6)	18% (13.2, 22.8)	22% (17.1, 26.9
Enugu	Lower Risk	50	Full Intervention	60% (53.5, 66.5)	82% (76.6, 87.4)	55% (47.4, 62.6)	80% (73.6, 86.4)	10% (5.6, 14.4)	8% (3.9, 12.1)

**Table 9:** a–d. Cross-tabulation results between specific variables (significant results in bold).

a. Cholera incidence and the practice of open defecation *			
Cholera Incidence	No Open Defecation	Open Defecation	Total
Pre-Intervention	53	35	88
Post-Intervention	48	44	92
Total	101	79	180
Chi-Square Test: Pearson χ^2^ (1) = 1.22, *p* = 0.271			
b. Cholera incidence and washing slaughtered animals in source waters			
Cholera Incidence	Washing Animals in Water	No Washing Animals in Water	Total
Pre-Intervention	42	64	106
Post-Intervention	48	26	74
Total	90	90	180
Chi-Square Test: Pearson χ^2^ (1) = 11.085, ***p* = 0.0019**			
c. Cholera incidence and hygiene education			
Cholera Incidence	Hygiene Education Pre-Test	Hygiene Education Post-Test	Total
No	41	46	87
Yes	46	46	92
Total	87	92	180
Chi-Square Test: Pearson χ^2^ (1) = 0.096, *p* = 0.757			
d. Use of chlorine and knowledge of cholera transmission			
Use of Chlorine	No Cholera Knowledge	Some Cholera Knowledge	Total
No	41	46	87
Yes	52	40	93
Total	93	87	180
Chi-Square Test: Pearson χ^2^ (1) = 1.482, *p* = 0.776			

* As estimated by surveys conducted by state health officials two years prior to this study.

**Table 10:** Results of logistic regression for cholera incidence (significant results in bold) *.

Variable	Odds Ratio (OR)	Standard Error	*p*-Value
WaSH program awareness	3.91	1.88	**0.005**
Access to healthcare resources	0.69	0.34	0.454
Open defecation	0.91	0.61	0.886
Belief in waterborne cholera	0.64	0.43	0.500
Use of chlorine for water treatment	0.70	0.33	0.453
Hygiene education	0.70	0.34	0.457
Constant	0.97	0.60	-

* Supplemental Table S4 presents a regression analysis of factors influencing cholera awareness and safe water practices (the effect size (Cramér’s V = 0.17) indicates a small effect of WaSH program awareness on cholera incidence. Additionally, the model fit statistics (Nagelkerke R^2^ = 0.20) suggest that the model explains about 20% of the variance in cholera incidence, indicating a moderate fit).

**Table 11:** Qualitative insights into traditional practices and infrastructural barriers to cholera prevention in Nigerian communities.

Theme	Key Findings	Implications for Cholera Prevention
Cultural Practices around Animal Washing	Washing slaughtered animals in drinking water sources was common, and many participants did not perceive it as a health risk.	The practice of washing animals in drinking water sources is a major cultural norm and must be addressed in future health education efforts.
Trust in Local Water Sources	Many community members trusted their local water sources because of long-standing familiarity not based on scientific knowledge of water contamination.	There is a need to shift community perceptions of water safety, emphasizing the risk of contamination and the importance of water treatment.
Barriers to Proper Sanitation	Limited access to sanitation facilities, lack of adequate waste disposal, and poor hygiene practices were frequently cited as major barriers.	Addressing infrastructural gaps and increasing the availability of latrines and waste management systems is crucial for cholera prevention.
Perceptions of Hygiene Education	Resistance to adopting new practices, especially when they conflicted with established customs.	Hygiene education efforts should be culturally tailored, addressing specific local practices and beliefs.
Impact of WaSH Programs	Higher levels of cholera knowledge and improved sanitation practices were observed in communities with strong WaSH program engagement.	WaSH programs can be effective in reducing cholera, but their reach and participation need to be expanded, especially in high-risk areas.
Role of Local Governance	Community leaders emphasized the need for local government support for sanitation infrastructure but noted a lack of resources and political will.	Strengthening local governance and inter-sectoral coordination is necessary to ensure the sustainability of cholera prevention programs.

## Data Availability

The raw data supporting the conclusions of this article will be made available by the authors on the request.

## Supplementary Materials

The following supporting information can be downloaded at https://www.mdpi.com/article/10.3390/ijerph22040483/s1. Box S1: Description of the Allen Foundation; Table S1: Educational Intervention Breakdown by Community; Table S2: Allen Foundation Survey; Table S3: Demographic Characteristics of Respondents by Region and Socio-Economic Status; Table S4. Regression Analysis of Factors Influencing Cholera Awareness and Safe Water Practices.
