# Supplementary material for: Exploring the Impact of Traditional Practices on Vibrio cholerae Outbreaks in Rural Nigerian Communities: A Field Study with Educational and Behavioral Interventions
Source: Int J Environ Res Public Health. 2025 Mar 24;22(4):483. doi: 10.3390/ijerph22040483 (PMC12027203; doi:10.3390/ijerph22040483)
Supplement: Supplementary file 1 [file ijerph-22-00483-s001.zip › ijerph-3495008 Box S1 Allen Foundation.pdf]

Box S1 *("The Allen Foundation, based in Nigeria, is a dynamic non-governmental organization committed to empowering young leaders through the provision of technical and vocational skills that are essential for thriving both locally and globally. Established with a focus on education, youth development, and health, the foundation aims to bridge critical gaps in knowledge and access to resources. Their work spans a variety of sectors, including health education, community mobilization, and advocacy for social change. By equipping young Nigerians with the skills and awareness needed to address societal and health challenges, the Allen Foundation is not only fostering individual growth but also driving community-driven development. Their programs are designed to create lasting impacts, particularly in underserved regions, by combining practical education with culturally relevant health interventions. This approach ensures that communities are not just informed, but actively engaged in sustainable solutions to local issues, including those related to water, sanitation, and hygiene (WaSH). Through these efforts, the Allen Foundation is helping to build a future where young people are equipped to lead in both their local environments and on the global stage" (Olalekan Oluwayomi, Founder, Allen Foundation, https://ng.linkedin.com/company/allen-foundation))
