# Supplementary material for: Exploring the Impact of Traditional Practices on Vibrio cholerae Outbreaks in Rural Nigerian Communities: A Field Study with Educational and Behavioral Interventions
Source: Int J Environ Res Public Health. 2025 Mar 24;22(4):483. doi: 10.3390/ijerph22040483 (PMC12027203; doi:10.3390/ijerph22040483)
Supplement: Supplementary file 1 [file ijerph-22-00483-s001.zip › ijerph-3495008 Table S1 Intervention Implementation.pdf]

Table S1: Educational Intervention Breakdown by Community

Component	Enugu State (Intervention)	Delta State (Minimal Intervention)	Ondo State (Partial Intervention)
**Data Collection & Community Engagement**	Key Informant Interviews, Focus Group Discussions (FGDs), and Town Hall Meetings conducted in all selected communities.	Basic cholera awareness pamphlets distributed. No formal FGDs or town hall meetings due to lack of a full intervention.	Key Informant Interviews, Focus Group Discussions (FGDs), and Town Hall Meetings conducted, but fewer communities engaged.
**Cholera Awareness Sessions**	Session 1: Introduction to Cholera and Transmission – conducted in all targeted communities.	Basic cholera awareness pamphlets distributed, but no formal education sessions held.	Session 1: Introduction to Cholera and Transmission – conducted, but some communities missed due to resource constraints.
	Session 2: Safe Water Practices – delivered to all communities.	No sessions conducted, though basic awareness was raised through pamphlets.	Session 2: Safe Water Practices – conducted, but fewer sessions due to resource limitations.
	Session 3: Cholera Symptoms and Dehydration Management – fully delivered.	No formal education on symptoms or dehydration management, only basic information in pamphlets.	Session 3: Cholera Symptoms and Dehydration Management – conducted, but not all the people in the community participated.
**Water Stewardship & Safe Practices**	Practical demonstrations on water treatment (boiling, chlorinating) conducted in all communities.	No hands-on demonstrations, only basic WASH awareness through pamphlets.	Practical demonstrations on water treatment – limited in scope, not all communities received hands-on learning.
	Focus on washing animals away from communal water sources.	No demonstrations, but pamphlets included some safe practices for water use.	Focus on washing animals away from communal water sources – partially conducted in select communities.
**Sanitation Practices & Latrine Construction**	Latrine construction and hygiene education fully implemented.	No latrine construction or sanitation education, minimal awareness through pamphlets.	Partial latrine construction in some communities due to limited resources, with hygiene education in selected areas.
	Hygiene education on latrine use and waste management in all communities.	No education on latrine use or waste management.	Hygiene education on latrine use and waste management – conducted in select communities.
**Community Mobilization**	Local leaders engaged in all communities to promote participation and reinforce health messages.	No formal community mobilization, only pamphlets distributed through community leaders.	Local leaders involved in a limited capacity, reinforcing messages in select communities.
**Printed Materials & SMS Reminders**	Flyers, posters, and SMS reminders distributed to all participants.	Basic pamphlets distributed, no SMS reminders or extensive materials.	Flyers, posters, and SMS reminders distributed in select communities.
**Behavioral Change Assessment (KAP Surveys)**	Pre- and post-surveys conducted to assess knowledge, attitudes, and practices (KAP) in all targeted communities.	Minimal KAP surveys conducted, improvements observed despite lack of formal intervention due to previous WASH efforts.	Pre- and post-surveys conducted, but limited in scope due to partial intervention.
**Challenges Encountered**	None significant, as intervention was implemented.	Limited formal intervention due to logistical challenges, but previous WASH efforts showed some impact.	Resource constraints, timing issues, and logistical problems limited full implementation.
**Follow-up & Monitoring**	Ongoing community visits to reinforce behavior change and monitor progress.	No follow-up or monitoring, though some improvement noted from prior WASH activities.	Limited follow-up and monitoring due to partial intervention.
