# Supplementary material for: Exploring the Impact of Traditional Practices on Vibrio cholerae Outbreaks in Rural Nigerian Communities: A Field Study with Educational and Behavioral Interventions
Source: Int J Environ Res Public Health. 2025 Mar 24;22(4):483. doi: 10.3390/ijerph22040483 (PMC12027203; doi:10.3390/ijerph22040483)
Supplement: Supplementary file 1 [file ijerph-22-00483-s001.zip › ijerph-3495008 Table S2 Survey Questions.pdf]

Supplemental Table S2 Allen Foundation Survey Questions

Water Use, Cholera Awareness, and Community Practices

Dear Respondent,

Please answer this questionnaire as honestly as possible. There is no wrong answer, so do not leave any questions unanswered. Let me assure you that your answers will be treated with utmost confidentiality.

Thank you

Allen Foundation, NGA

Part 1: Respondent Profile

Please provide the necessary information or put a check mark (/) next to the item that corresponds to your response.

1. **Name**: __________________________
2. **Age**:
   - o 14-17 years
   - o 18-24 years
   - o 25-35 years
   - o 35-44 years
   - o 45 years and above
3. **Sex**:
   - o Male
   - o Female
4. **Educational Background**:
   - o No Education
   - o Primary School Level
   - o Secondary School Level
   - o Tertiary Level (University/College)
5. **Income Level (in USD per year)**:
   - o Less than $100 (N500 - N1,000)
   - o $100 - $300 (N1,001 - N5,000)
   - o $300 - $600 (N5,001 - N15,000)
   - o $600 - $1,200 (N15,001 - N25,000)
   - o More than $1,200 (N25,001 - N30,000)

Part 2: Behavior and Perceptions Regarding Water Sources

Instructions: Below are a series of statements about community practices related to water sources and sanitation. Please read each statement carefully and indicate how much you agree or disagree using the following scale:

4 = Strongly Agree
3 = Agree
2 = Disagree
1 = Strongly Disagree

Statement	4 (Strongly Agree)	3 (Agree)	2 (Disagree)	1 (Strongly Disagree)
If I see someone washing slaughtered animals in a river or stream, I will say something to them.	☐	☐	☐	☐
If I see someone bathing in the water source, I will inform them that it could be dangerous.	☐	☐	☐	☐
I believe that washing slaughtered animals in water sources can make the water unsafe for drinking.	☐	☐	☐	☐
I would speak out if I saw someone washing their clothes in water sources that people drink from.	☐	☐	☐	☐
I believe that vomiting and diarrhea are signs of cholera.	☐	☐	☐	☐
I believe that drinking untreated water can lead to cholera.	☐	☐	☐	☐
I know where to report symptoms of cholera to the health department.	☐	☐	☐	☐
I know what to do to prevent dehydration if someone gets cholera.	☐	☐	☐	☐
I believe that open defecation can contribute to cholera outbreaks.	☐	☐	☐	☐

Part 3: Knowledge and Awareness of Cholera

Instructions: Please answer the following questions.

1. **What do you understand by cholera?**
   (Open-ended question to assess community knowledge on cholera.)

2. **How do you believe cholera spreads in your community?**
   (Open-ended question to capture any misconceptions or correct knowledge about cholera transmission.)
3. **What are the symptoms of cholera?**
   (Open-ended question to understand if the community knows the warning signs.)
4. **What should you do if you suspect someone has cholera?**
   (Open-ended question to assess actions taken by the community in response to cholera symptoms.)

Part 4: Practices Regarding Water Use and Hygiene

Instructions: Answer the following questions about your water use and sanitation practices.

1. **Do you wash slaughtered animals in water sources (rivers, streams, wells)?**
   - Yes
   - No
   - If yes, please explain why: _______________
2. **Do you treat your drinking water before consumption?**
   - Yes
   - No
   - If yes, how do you treat it? (e.g., boiling, filtering, etc.): _______________
3. **Do you collect water from the well or nearby river/stream?**
   - Well
   - River/Stream
   - Both
   - Other (please specify): ___________
4. **Why do you prefer the water source you use?**
   (Open-ended questions to understand preferences and reasons behind them.)

Part 5: Water Infrastructure and Community Practices

Instructions: Please read the statements below and indicate your agreement using the scale provided.

Statement	4 (Strongly Agree)	3 (Agree)	2 (Disagree)	1 (Strongly Disagree)
I trust the water from the well more than the water from the river.	☐	☐	☐	☐
The well water is often unsafe or unpleasant to drink.	☐	☐	☐	☐

Statement	4 (Strongly Agree)	3 (Agree)	2 (Disagree)	1 (Strongly Disagree)
The river water is the main source of drinking water for my household.	☐	☐	☐	☐
I prefer to travel far to collect clean water rather than using the local well.	☐	☐	☐	☐
I believe the community should work together to protect local water sources from contamination.	☐	☐	☐	☐

Part 6: Suggestions for Improvement

1. **What changes do you think should be made to improve the quality of water in your community?**
   (Open-ended question to understand community ideas for water safety.)
2. **What should the government do to prevent cholera outbreaks in your community?**
   (Open-ended question to understand local expectations from authorities.)
3. **What can the community do to improve hygiene and prevent the spread of cholera?**
   (Open-ended questions to gather community-driven solutions.)
4. **If there were an awareness campaign on cholera prevention, what methods would be most effective? (For example, posters, community meetings, radio, etc.)**
   (Open-ended question for community engagement ideas.)

Thank you for your time and responses!
