# Supplementary material for: Exploring the Impact of Traditional Practices on Vibrio cholerae Outbreaks in Rural Nigerian Communities: A Field Study with Educational and Behavioral Interventions
Source: Int J Environ Res Public Health. 2025 Mar 24;22(4):483. doi: 10.3390/ijerph22040483 (PMC12027203; doi:10.3390/ijerph22040483)
Supplement: Supplementary file 1 [file ijerph-22-00483-s001.zip › ijerph-3495008 Table S3 Demographic Characteristics (1).pdf]

Table S3. Demographic Characteristics of Respondents by Region and Socio-Economic Status

Characteristic	Delta (High-Risk)	Ondo (Mixed-Risk)	Enugu (Low-Risk)	Overall
**Gender Distribution**				
Female	32 (17.8%)	22 (12.2%)	21 (11.6%)	85 (47%)
Male	34 (19%)	31 (17.2%)	30 (16.6%)	95 (53%)
**Age Distribution**				
14-17 years	7 (3.9%)	9 (5%)	6 (3.3%)	22 (12.0%)
18-24 years	13 (7.2%)	11 (6.1%)	19 (10.5%)	43 (23.9%)
25-35 years	27 (15%)	22 (12.2%)	24 (13.3%)	73 (41.0%)
35-44 years	7 (3.9%)	3 (1.7%)	2 (1.1%)	12 (7.0%)
45 years and above	10 (5.5%)	14 (7.7%)	7 (3.8%)	30 (16.6%)
**Educational Background**				
No Education	9 (5.0%)	8 (4.4%)	12 (6.6%)	29 (16.0%)
Primary School Level	32 (17.7%)	23 (12.8%)	21 (11.7%)	76 (42.2%)
Secondary School Level	28 (15.6%)	25 (13.9%)	22 (12.2%)	75 (41.7%)
**Income Level (Annual)**				
N500-N1,000 per annum	18 (10%)	21 (11.6%)	21 (11.6%)	60 (33%)
N1,001-N5,000 per annum	10 (6.0%)	12 (6.6%)	14 (7.7%)	36 (20.0%)
N5,001-N15,000 per annum	3 (2.0%)	8 (4.0%)	9 (5.0%)	20 (11.0%)
N15,001-N25,000 per annum	12 (7.0%)	8 (4.0%)	14 (8.0%)	34 (19.0%)
N25,000-N30,000 per annum	8 (4.4%)	7 (3.8%)	15 (8.0%)	30 (17.0%)

Notes:

1. **Income Determination:** The income brackets were self-reported and based on household estimates. The specific values for annual income were determined by asking participants to approximate their household's income over the past year, which was then categorized into the specified income ranges. The reported income ranges are approximate and may have been subject to fluctuations due to currency instability (Nigerian Naira) during the study period.
2. **Geographic Risk Classification:** The states were categorized into high-risk (Delta), mixed-risk (Ondo), and low-risk (Enugu) based on previous cholera incidence data and consultations with local health authorities. These classifications help contextualize the risk exposure and potential vulnerability of the populations to cholera outbreaks.
3. **Socio-Economic Conditions:** Socio-economic data such as income and education levels were used to explore potential confounding factors affecting cholera knowledge and safe water practices. These factors are crucial in understanding the variation in awareness and behavior across the study populations, as individuals in lower income brackets or with lower education levels may face additional barriers to adopting cholera-prevention behaviors.

Commented [SA1]: Maybe discussion?
