# Supplementary material for: Exploring the Impact of Traditional Practices on Vibrio cholerae Outbreaks in Rural Nigerian Communities: A Field Study with Educational and Behavioral Interventions
Source: Int J Environ Res Public Health. 2025 Mar 24;22(4):483. doi: 10.3390/ijerph22040483 (PMC12027203; doi:10.3390/ijerph22040483)
Supplement: Supplementary file 1 [file ijerph-22-00483-s001.zip › ijerph-3495008 Table S4 Regression Analysis.pdf]

Table S4. Regression Analysis of Factors Influencing Cholera Awareness and Safe Water Practices

Variable	Odds Ratio (OR)	95% Confidence Interval (CI)	p-value	Interpretation
**Gender**	1.15	0.85-1.56	0.35	No significant effect on cholera awareness.
**Age**	1.30	1.05-1.65	0.02*	Younger age groups more likely to report safe water practices.
**Education Level**	1.45	1.20-1.75	0.01*	Higher education levels associated with better cholera knowledge.
**Income Level (N1,001-N5,000)**	1.32	1.05-1.66	0.04*	Moderate income group more likely to adopt safe practices.
**Income Level (N5,001-N15,000)**	1.08	0.75-1.52	0.68	No significant effect for this group.
**Safe Water Access**	2.12	1.75-2.59	<0.001**	Safe water access significantly improves safe practices.
**Community Leader Involvement**	1.78	1.42-2.23	0.003**	Active involvement of community leaders significantly improves hygiene practices.
**Intervention Exposure**	1.50	1.12-1.99	0.005**	Intervention exposure has a positive impact on both cholera knowledge and safe water practices.

Notes:

1. **Confidence Intervals & Statistical Significance:** All variables were adjusted for confounding socio-economic and geographic factors in the regression model. The results indicate the extent to which socio-economic factors, like income and education, influenced participants' cholera awareness and safe water practices.
2. **Income and Education Variables:** Specifically, the study accounted for both income level and education, as they have been found to strongly correlate with access to resources and knowledge regarding cholera prevention. The fluctuations in income, particularly with exchange rate volatility, were considered by grouping income levels into discrete categories rather than exact amounts.
3. **Pre vs. Post-Survey Comparison:** Incidence measurements were based on pre- and post-survey data, which compared the “always used safe practices before” and “after intervention” responses, as reflected in the regression model. This allows for a comparison of behavior changes over time.
